# Overcoming Drug Loading and Dosage Volume Challenges of Adsorption-Solidified SNEDDS by pH-Modulation Strategy: Atorvastatin Calcium and Glibenclamide as Model Drugs

**DOI:** 10.3390/pharmaceutics18080942

**Published:** 2026-07-30

**Authors:** Abdelrahman Y. Sherif, Mohammad A. Altamimi

**Affiliations:** Department of Pharmaceutics, College of Pharmacy, King Saud University, Riyadh 11451, Saudi Arabia; maltamimi@ksu.edu.sa

**Keywords:** self-nanoemulsifying drug delivery system, pH modulation, sodium carbonate, Syloid, atorvastatin calcium, glibenclamide

## Abstract

**Background**: Adsorption-based solidification is a solvent-free route to prepare a solid form of self-nanoemulsifying drug delivery systems (SNEDDS). However, the limited drug loading and the low bulk density of the porous carrier restrict its pharmaceutical applicability. This study developed a pH-modulated SNEDDS in which sodium carbonate modulates the pH of the formulation microenvironment. Atorvastatin calcium and glibenclamide were used as high-dose and low-dose weakly acidic model drugs. **Methods**: The SNEDDS components were selected by solubility and emulsification screening. Sodium carbonate was incorporated as the pH-modulating agent, and liquid formulations were solidified by adsorption onto Syloid. The formulations were characterized by FTIR, PXRD, and SEM, and evaluated by an in vitro dissolution study. **Results**: The selected liquid SNEDDS (L-SNEDDS) consisted of polysorbate 80, polyethylene glycol 400, and glyceryl monocaprylate. Sodium carbonate increased the microenvironmental pH from 5.31 to 6.83. This increased drug loading by approximately 2.0-fold for atorvastatin calcium and 3.0-fold for glibenclamide. FTIR showed no chemical interaction between the components. SEM confirmed adsorption within the porous carrier, whereas PXRD showed no detectable drug crystallinity. The increased loading reduced the number of capsules required per dose from two to one for atorvastatin calcium and from four to one for glibenclamide. In vitro dissolution confirmed that pH modulation did not compromise drug dissolution despite the reduced SNEDDS content per dose. **Conclusions**: pH modulation with sodium carbonate enabled single-capsule dosing and provided a solvent-free route to boost drug loading for the two investigated model drugs.

## 1. Introduction

A Self-nanoemulsifying Drug Delivery System (SNEDDS) is a thermodynamically stable isotropic mixture that forms nanoemulsion droplets upon dispersion in aqueous media with mild agitation [1]. It has been widely utilized in the literature to boost the bioavailability of hydrophobic molecules with low water solubility [2]. However, formulation leakage from capsules during storage hampers the pharmaceutical application of liquid SNEDDS (L-SNEDDS) [3]. Consequently, the transformation into solid dosage forms has attracted substantial research interest to enhance their pharmaceutical applicability [4,5].

Several approaches have been reported for converting L-SNEDDS into solid SNEDDS (S-SNEDDS). This was achieved using spray drying, freeze-drying, melt granulation, hot-melt extrusion, and adsorption approaches [6,7]. Among these, adsorption onto a solid carrier has been widely applied owing to the expected reduction in production costs [8]. This is attributed to its simpler preparation method, in which the ingredients are mixed for a short period of time to obtain the solid SNEDDS formulation [9]. Moreover, solvent-free processing reduces production time by eliminating the time-consuming solvent evaporation step in other methods [10]. Furthermore, pharmaceutical processing at room temperature makes adsorption suitable for thermosensitive molecules [11].

A solid form of SNEDDS using this approach is achieved by physically adsorbing the liquid formulation onto the high surface area of porous materials [12]. Unfortunately, the adsorption-based solidification approach presents inherent challenges that limit its practical applicability. Firstly, the limited solubility of the drug in the SNEDDS formulation increases the required dose volume. Moreover, the low density of the used porous carriers increases the total volume of the dosage unit that needs to be filled within the capsule [13]. Therefore, there is an insistent demand to resolve these issues to enhance the pharmaceutical applicability of the adsorption approach.

The lower acceptable limit for the ratio of drug-loaded liquid SNEDDS to the adsorbent was usually set at an equal weight ratio to ensure complete solidification of the liquid SNEDDS [14,15,16]. Therefore, increasing the drug-loading capacity in a liquid formulation is preferable to reducing the total volume of solidified SNEDDS. This is anticipated to decrease the volume of the dosage form containing the therapeutic dose, allowing it to be filled into a single capsule.

The drug loading efficiency of weakly basic drugs could be enhanced using free fatty acid components like capric and oleic acid [17]. Despite their lipophilic nature, weakly acidic drugs usually exhibit low solubility in SNEDDS formulations. This is ascribed to the presence of drugs in unionized form, which reduces their solubility. Consequently, pH modulation of the SNEDDS microenvironment represents an attractive approach to boost drug solubility within the formulation. The integration of the pH-modulation agent into SNEDDS is expected to increase the microenvironment pH above the pKa of weakly acidic drugs. This is expected to enhance ionization and drug loading of these agents within the SNEDDS formulation [18].

**Figure 1:**
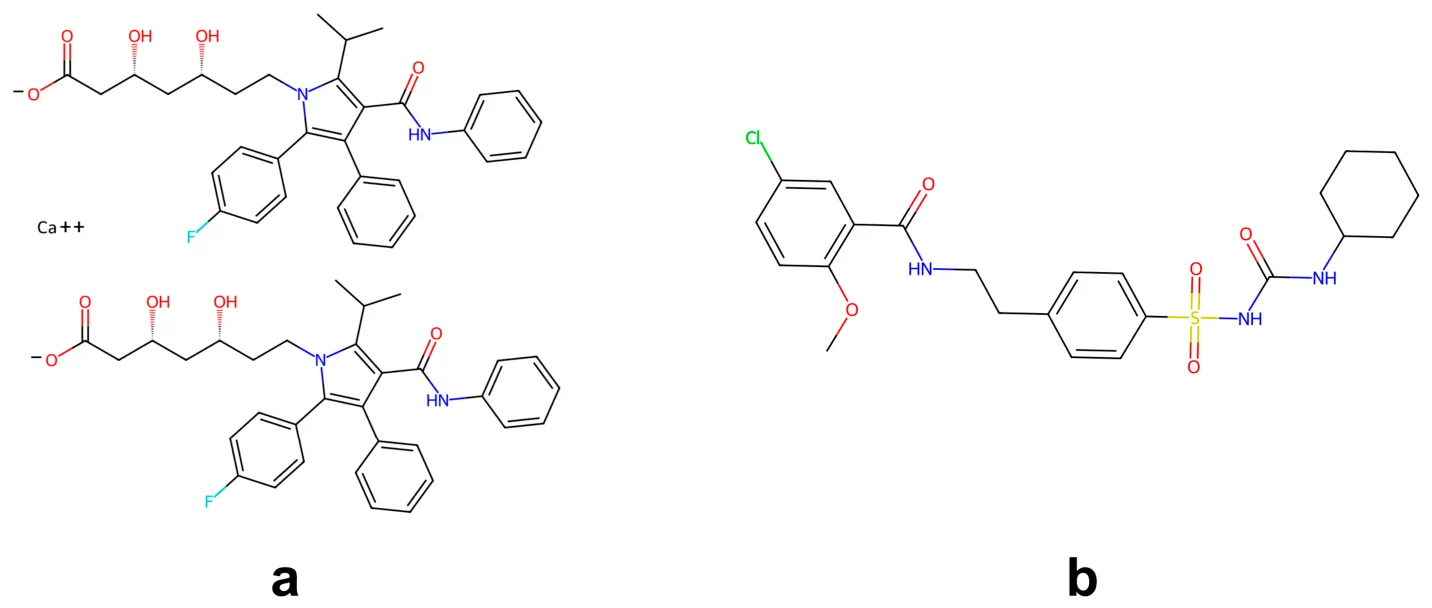
Chemical structure of (**a**) atorvastatin calcium and (**b**) glibenclamide.

Atorvastatin calcium and glibenclamide (Figure 1) were selected as models of weak acid drugs to represent high and low doses, respectively. Atorvastatin calcium is a widely prescribed HMG-CoA reductase inhibitor used to manage hypercholesterolemia, whereas glibenclamide is a second-generation sulfonylurea used to control type 2 diabetes mellitus. Both drugs belong to Class II drugs (low solubility) that exhibit low and variable oral bioavailability [19,20]. It should be noted that although atorvastatin calcium exhibits appreciable solubility in intestinal media, several in vivo studies have demonstrated that its formulation as an SNEDDS markedly enhances oral bioavailability compared with the raw drug. This confirms that the benefit of the SNEDDS is not limited to overcoming the equilibrium solubility limit [21,22].

The present study aimed to develop a pH-modulated SNEDDS to overcome the limitations of low drug loading and high dosing volume associated with adsorption-based solid SNEDDS. The approach is based on incorporating sodium carbonate as an alkalizing agent to raise the formulation’s microenvironmental pH above the drug’s pKa. This enhances the ionization of the weakly acidic drug and increases its loading within the SNEDDS formulation. Moreover, the sodium counterion increases the loading capacity for the loaded drug through association with the ionized drug. Atorvastatin calcium and glibenclamide were selected as high-dose and low-dose weakly acidic model drugs to represent the range of doses encountered in adsorption-based solid SNEDDS. The specific objectives were to establish the effect of the pH-modulating agent on the microenvironmental pH and drug loading. The impact of pH modulation on the dosage volume and the in vitro dissolution of the two model drugs was also assessed.

## 2. Materials and Methods

### 2.1. Materials

Riyadh Pharma (Riyadh, Saudi Arabia) and Saudi Pharmaceutical Industries and Medical Appliances Corp. (Qassim, Saudi Arabia) provided Atorvastatin calcium (ATV) and glibenclamide (GLB), respectively. Loba Chemie (Mumbai, India) supplied Polysorbate 80 (Tween 80). Merck-Schuchardt OHG (Hohenbrunn, Germany) supplied both Polysorbate 60 (Tween 60) and Polysorbate 85 (Tween 85). BASF (Ludwigshafen, Germany) provided the cosurfactant ingredient polyethylene glycol 400 (Kollisolv PEG 400). Gattefosse (Saint-Priest, France) provided glyceryl monolinoleate (Maisine 35-1) and Glyceryl monooleate (Peceol). Avonchem (Macclesfield, Cheshire, UK) provided oleic acid and olive oil, while Sasol Germany GmbH (Witten, Germany) provided glyceryl monocaprylate (Imwitor 308). Cremer Oleo GmbH & Co. KG (Witten, Germany) supplied medium-chain triglycerides (Miglyol 810 N). Winlab (Market Harborough, UK) supplied arachis oil.

### 2.2. UPLC Method for Atorvastatin Calcium and Glibenclamide Analysis

Quantification of glibenclamide and atorvastatin calcium was performed using previously developed ultra-performance liquid chromatography (UPLC) methods. The atorvastatin calcium and glibenclamide concentrations in the analyzed samples were quantified using a Dionex™ UPLC system (Thermo Scientific, Bedford, MA, USA). Chromatographic separation was achieved using Acquity UPLC BEH C18 columns. Atorvastatin calcium and glibenclamide concentrations were calculated from linear calibration curves. Detailed chromatographic conditions for each analyte are summarized in Table 1.

### 2.3. Solubility

The equilibrium solubility of atorvastatin calcium and glibenclamide was estimated in the individual SNEDDS components and in the selected formulations. The solubility of each agent was estimated independently for each component. An excess amount of atorvastatin calcium and glibenclamide was placed inside a glass vial along with the tested agent and mixed using a 5-position magnetic stirrer (IKA-Werke GmbH & Co. KG, Staufen, Germany) at 1000 rpm for 1 day at a controlled room temperature of 23 ± 2 °C. Each sample was then centrifuged using a Hettich Mikro 120 microcentrifuge (Andreas Hettich GmbH & Co. KG, Tuttlingen, Germany) at 13,000 rpm for 10 min to separate the undissolved drug. A precisely weighted aliquot of the clear supernatant was quantitatively diluted with acetonitrile to extract the dissolved drug into a homogeneous solution suitable for chromatographic analysis. The drug concentration was then determined by UPLC as described in Section 2.2.

### 2.4. Emulsification Study

The emulsification efficiency of polysorbate 85, polysorbate 80, polysorbate 60, sorbitan monooleate, and sorbitan monolaurate for glyceryl monocaprylate (the selected oil phase) was evaluated to identify the optimal surfactant for the SNEDDS. Each surfactant was blended with glyceryl monocaprylate and mixed until a homogeneous formulation was obtained. Each homogeneous mixture was then dispersed by dilution 1:1000 in Milli-Q water and stirred. The percentage transmittance of the resulting dispersions was measured at 638 nm against Milli-Q water as a blank using a PD-303UV spectrophotometer (APEL, Saitama, Japan).

### 2.5. Preparation of SNEDDS Formulation

The L-SNEDDS vehicle was prepared by mixing polysorbate 80, polyethylene glycol 400, and glyceryl monocaprylate at a fixed weight ratio of 4:3:3. The oil phase and surfactant were selected based on drug solubility and emulsification studies. For the pH-modulated formulations (PM-L-SNEDDS), sodium carbonate was incorporated into the L-SNEDDS at a concentration of 5 mg/g. Drug loading was set below the measured saturation solubility in each formulation to avoid supersaturation and the risk of drug precipitation on storage. The model drugs were then loaded into the corresponding formulation: atorvastatin calcium at 80 mg/g in the L-SNEDDS and 160 mg/g in the PM-L-SNEDDS, and glibenclamide at 5 mg/g in the L-SNEDDS and 15 mg/g in the PM-L-SNEDDS. Finally, the drug-loaded L-SNEDDS and PM-L-SNEDDS were solidified by mixing with silicon dioxide (Syloid) at a 1:1 weight ratio to obtain a free-flowing solid formulation.

### 2.6. Droplet Size Analysis of the Aqueous Dispersion of SNEDDS

The droplet size and polydispersity index (PDI) of the dispersed L-SNEDDS and PM-L-SNEDDS formulations were determined by dynamic light scattering using a Zetasizer Nano ZS (Malvern Panalytical, Malvern, UK). Each formulation was dispersed in distilled water at a ratio of 1:250 and stirred. The resulting dispersion was transferred into a disposable cuvette and equilibrated at 25 °C before measurement.

### 2.7. pH Measaurement

The microenvironmental pH of L-SNEDDS and PM-L-SNEDDS formulations was determined using a calibrated pH meter (HI 2211 pH/ORP meter, Hanna Instruments, Woonsocket, RI, USA). The electrode was immersed directly into the tested formulation at room temperature, and the pH value was recorded after the reading had stabilized. All measurements were performed in triplicate and are expressed as mean ± SD.

### 2.8. SEM

Scanning electron microscopy (SEM) was used to examine the surface morphology of both pure drugs (atorvastatin calcium and glibenclamide), the Syloid adsorbent, and the solidified SNEDDS formulations (ATV-S-SNEDDS, ATV-PM-S-SNEDDS, GLB-S-SNEDDS, and GLB-PM-S-SNEDDS). This analysis was performed to investigate the incorporation of each drug within the formulation matrix and to assess the effect of the loaded SNEDDS on the adsorbent carrier. Before imaging, the samples were mounted on aluminium stubs and sputter-coated with a thin gold layer for 60 s at 20 mA under an argon atmosphere using a Q150R sputter coater (Quorum Technologies Ltd., East Sussex, UK). The samples were then examined on a Zeiss EVO LS10 microscope (Carl Zeiss, Cambridge, MA, USA) operated in high-vacuum mode, using the secondary electron detector at an accelerating voltage (EHT) of 20 kV and a working distance of 9.5 mm.

### 2.9. FTIR

A PerkinElmer Spectrum-100 Spectrometer (Waltham, MA, USA) was used to obtain Fourier transform infrared (FTIR) spectra of the tested agents. The tested agents were scanned over the range 750–4000 cm^−1^ after placement on the diamond ATR crystal and application of pressure with the sampling accessory. Spectrum software version 6.3.5 (PerkinElmer, Inc., Waltham, MA, USA) was used to acquire and process spectra.

### 2.10. PXRD

The crystalline-versus-amorphous character of atorvastatin calcium and glibenclamide was determined by powder X-ray diffractometry (PXRD) on a Rigaku Ultima IV goniometer (Rigaku Corporation, Tokyo, Japan) operated in coupled 2θ/θ geometry. A copper target supplied Cu Kα radiation (λ = 1.5406 Å), the tube being energized at 40 kV and 30 mA. Diffracted photons were registered with a scintillation counter seated behind a fixed diffracted-beam monochromator. Neither an incident-beam monochromator nor a β-filter was interposed in the optical path. Beam divergence and parasitic scatter were regulated by a 2/3° divergence slit, a 10 mm height-limiting slit, a 2/3° scatter slit, and a 0.3 mm receiving slit. Each specimen was finely comminuted to a uniform powder and packed level into the wells of a ten-position automated sample changer. It was interrogated at ambient temperature under continuous scanning across a 3–30° 2θ window, the goniometer advancing at 1.0° min^−1^ with a 0.1° 2θ sampling step and a nil theta offset.

### 2.11. In Vitro Dissolution Study

The dissolution profiles of atorvastatin calcium and glibenclamide were evaluated using a type II dissolution instrument (LOGAN Inst. Corp., Somerset, NJ, USA). The tested formulations (L-SNEDDS, PM-L-SNEDDS, S-SNEDDS, and PM-S-SNEDDS) were placed in the required number of hard gelatin capsules before the experiment, at a dose equivalent to 40 mg of atorvastatin calcium or 5 mg of glibenclamide. Moreover, phosphate buffer (pH 6.8, 900 mL) was placed in dissolution vessels, and the heating temperature was set to 37 ± 0.5 °C to simulate physiological conditions. The paddle speed was set at 50 rpm to simulate peristaltic movement. At the beginning of the experiment, the capsules were placed within wires with a cylindrical shape and immersed in the preheated dissolution media. At the following time points (5, 10, 15, 30, 45, and 60 min), 1.5 mL was withdrawn through a syringe fitted with a filter from the dissolution media and analyzed by the developed UPLC method.

### 2.12. Use of Generative AI

Certain portions of this manuscript were drafted and/or refined with the assistance of Claude (a large language model developed by Anthropic, San Francisco, CA, USA). However, the authors held full responsibility for the overall conceptualization and findings.

## 3. Results and Discussion

### 3.1. UPLC Quantification of Atorvastatin Calcium and Glibenclamide

**Figure 2:**
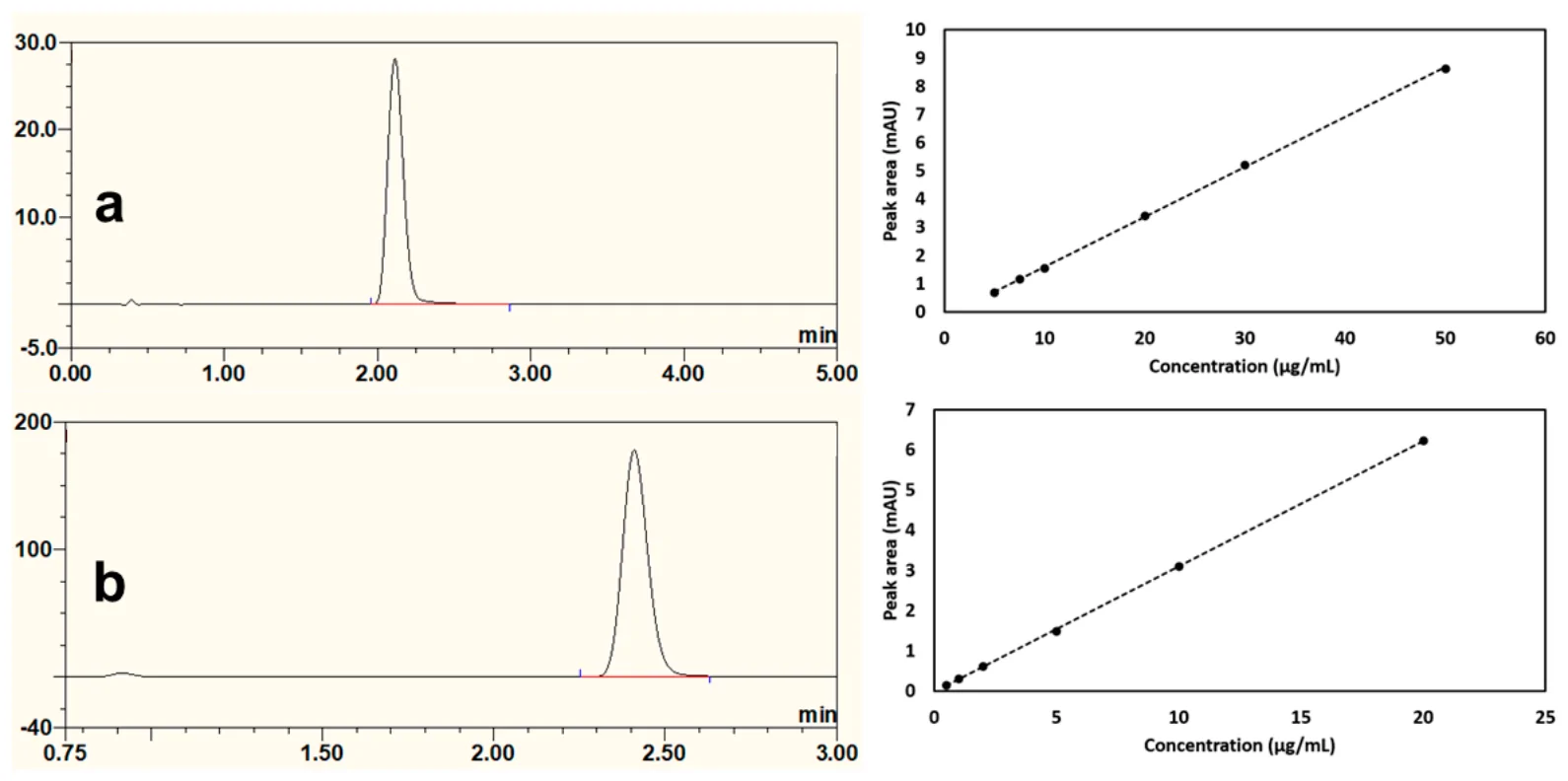
Representative UPLC chromatograms and corresponding calibration curves of (**a**) atorvastatin calcium and (**b**) glibenclamide.

The developed UPLC methods were used to quantify atorvastatin calcium and glibenclamide in the tested samples. Calibration curves of atorvastatin calcium and glibenclamide were linear over 5–50 µg/mL and 0.5–20 µg/mL, with coefficients of determination (R^2^) of 0.9997 and 0.9999, respectively. Regression analysis produced the equations: peak area = 0.1772 × concentration − 0.1744 for atorvastatin calcium, and peak area = 0.3120 × concentration − 0.0173 for glibenclamide. These calibration curves confirmed a linear relationship across the tested ranges (Figure 2). The percentage recovery ranged from 96.44% to 101.49% for atorvastatin calcium and from 96.53% to 105.13% for glibenclamide. Atorvastatin calcium eluted at a retention time of 2.13 min, and glibenclamide at 2.43 min (Figure 2).

### 3.2. Drug Soluability in Oils

**Figure 3:**
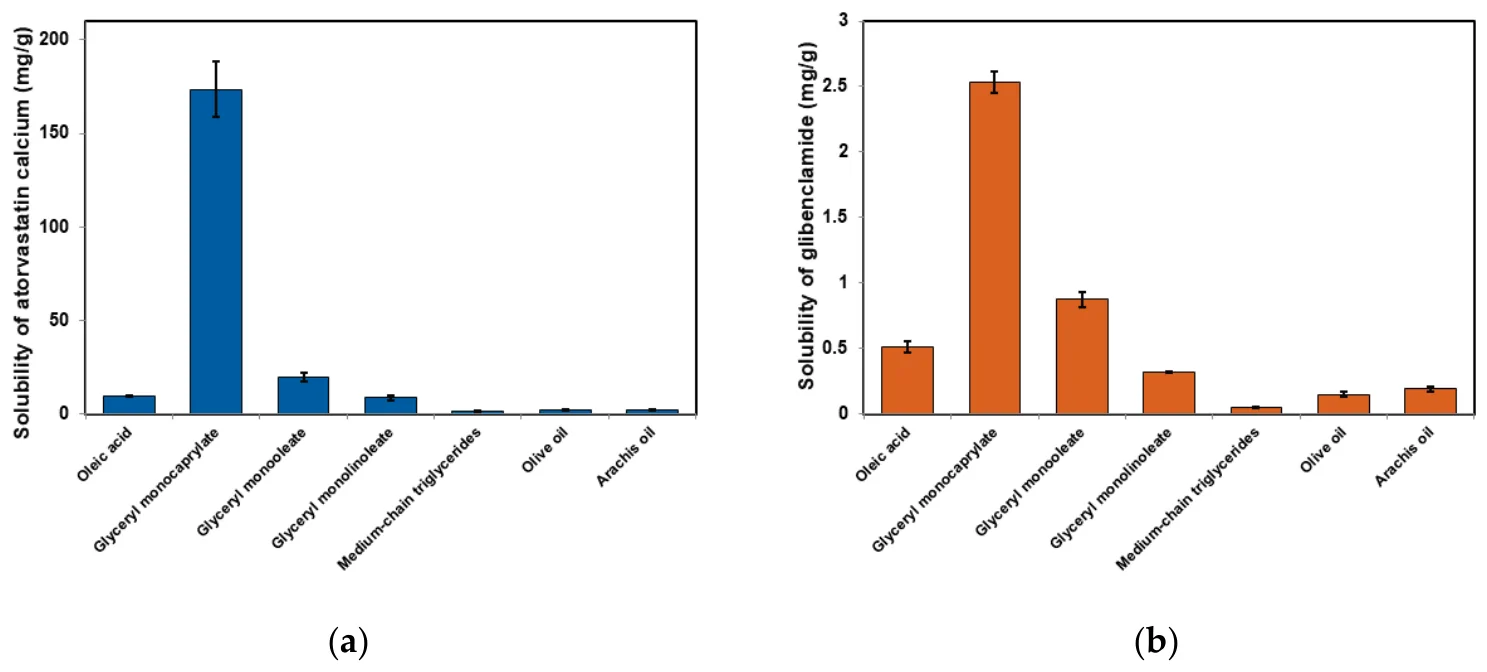
Solubility of (**a**) atorvastatin calcium and (**b**) glibenclamide in the screened oils.

Figure 3 presents atorvastatin calcium and glibenclamide solubility in the selected oils. The current results showed that the solubility of both drugs depends on the degree of esterification, the fatty acid chain length, and the degree of saturation. Both drugs showed higher solubility in monoglycerides than in free fatty acids and triglycerides. Maximum atorvastatin calcium and glibenclamide solubility was observed in glyceryl monocaprylate (glycerol monocaprylate; medium chain monoglyceride) with values of 173.38 ± 14.85 and 2.53 ± 0.082 mg/g, respectively.

The high drug solubility observed in glyceryl monocaprylate could be attributed to the presence of two free hydroxyl groups, the shorter chain length, and the degree of saturation. It has been reported that atorvastatin forms a hydrogen bond through the amide and hydroxyl groups [23,24], while glibenclamide forms a hydrogen bond through carbonyl and sulfonyl groups [25]. Therefore, the presence of two unesterified hydroxyl groups on the glycerol backbone allows hydrogen bonding with the polar functional groups of drug molecules and increased drug solubility [26]. However, the superior performance of oils containing shorter chain lengths (glyceryl monocaprylate) could be ascribed to the abundance of ester moieties per gram compared to those containing longer chain lengths (glyceryl monooleate and glyceryl monolinoleate). Consequently, the abundance of hydroxyl groups in glyceryl monocaprylate, as determined by molar calculation, could contribute to its superior solubilization capacity [27]. Moreover, oils containing monounsaturated fatty acids (glyceryl monooleate) showed higher solubilization power than polyunsaturated oils (glyceryl monolinoleate). These findings are consistent with prior literature, which reported higher drug solubility in saturated oils than in unsaturated oils [28].

However, the superior solubilization power of free fatty acids compared to triglycerides could be ascribed to the presence of free carboxylic acid, which is reported to form hydrogen bonds with corresponding groups in both drugs [29]. This agrees with current findings, which show the lowest drug solubility in triglycerides for both drugs owing to esterification of all hydroxyl groups. This reduces hydrogen-bond formation and lowers drug solubility in triglyceride-containing oils. In agreement with current results, Rane et al. demonstrated a linear relationship between the oil’s hydroxyl group content and drug solubility [30].

Finally, glyceryl monocaprylate was selected as the optimal oil component for preparing the SNEDDS formulation owing to its high measured drug solubility. This provides outstanding advantages in terms of pharmaceutical preparation and in vivo performance. For the pharmaceutical preparation aspect, increasing drug solubility reduces the total formulation volume required to deliver a therapeutic dose to patients [31]. From the in vivo performance viewpoint, using oil with high drug solubility could increase the chance of drug association with formed nanoemulsion droplets in vivo and enhance drug bioavailability [32].

### 3.3. Emulsification of Surfactants

The prepared mixtures consisting of surfactant (polysorbate 85, polysorbate 80, polysorbate 60, sorbitan monooleate, and sorbitan monolaurate) and glyceryl monocaprylate were dispersed in aqueous media to investigate their emulsification effects. The sorbitan monooleate and sorbitan monolaurate were excluded from the study because they failed to produce an emulsion system with the selected oil (glyceryl monocaprylate). However, the transmittance percentage for the dispersed emulsion system containing polysorbate surfactants was recorded and displayed in Table 2. Polysorbate 80 was selected as a surfactant component owing to the measured emulsification power for glyceryl monocaprylate.

### 3.4. Physicochemical Characterization of Liquid SNEDDS Formulations

The prepared L-SNEDDS formulation was mixed with sodium carbonate at a 5 mg/g loading capacity to prepare a pH-modulated liquid SNEDDS formulation. The two prepared formulations were subjected to various assessments to investigate the effects of a pH-modulating agent on their physicochemical properties and drug-loading capacity.

#### 3.4.1. Physical Appearance and Droplet Size of the Aqueous Dispersion of SNEDDS

**Figure 4:**
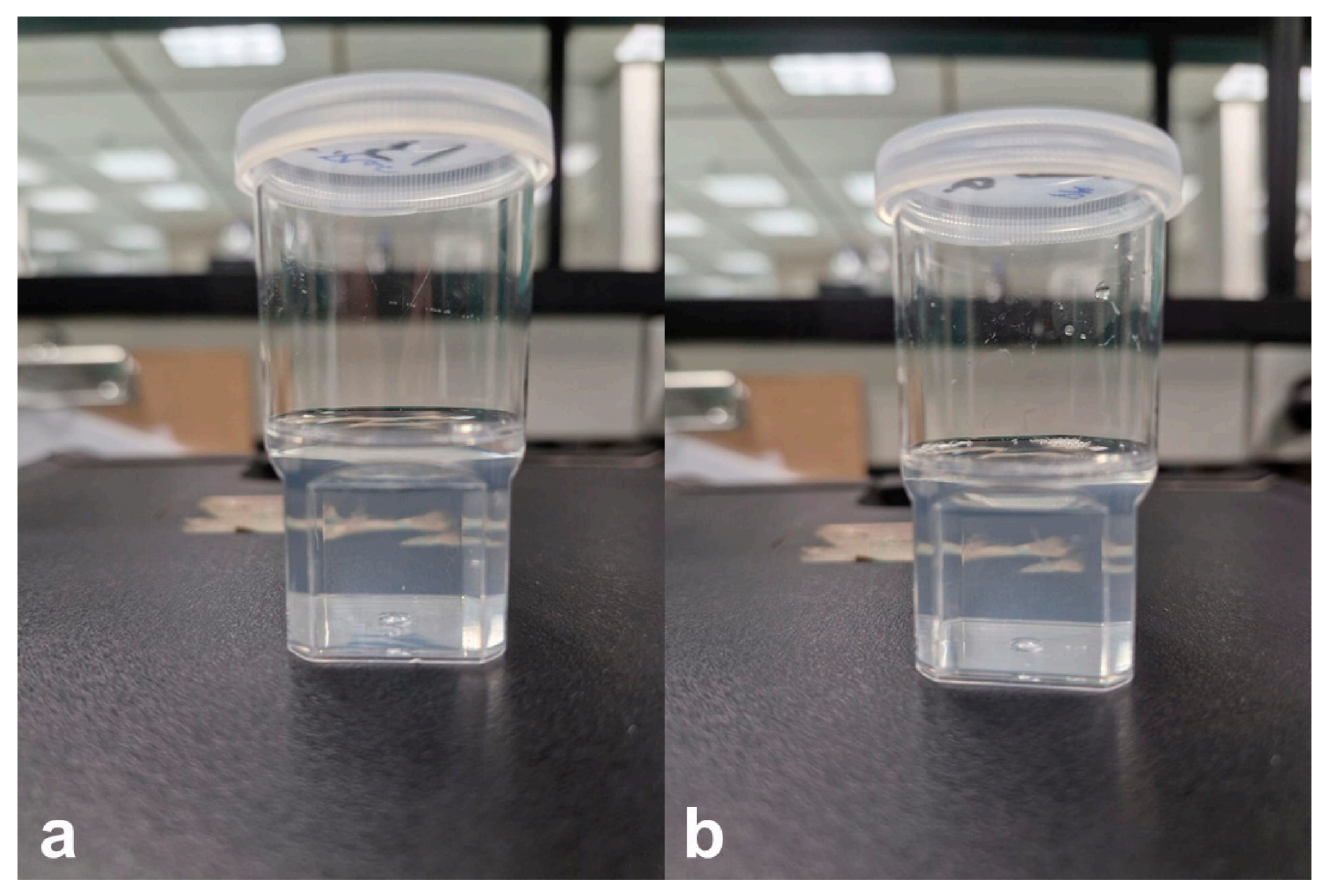
Physical appearance of the aqueous dispersions of (**a**) liquid SNEDDS and (**b**) pH-modulated liquid SNEDDS following dilution with distilled water at a ratio of 1:250.

The two prepared formulations were dispersed in distilled water, and the resulting self-emulsifying dispersions are shown in Figure 4. The images show that aqueous dispersions of both formulations were clear. This indicates successful emulsification of the dispersed system, with no negative impact on its appearance due to the pH-modulating agent. Moreover, the droplet size and polydispersity index of the dispersed L-SNEDDS and PM-L-SNEDDS formulations are 198.5 ± 12.18 nm and 252.8 ± 5.65 nm, and PDI values of 0.536 ± 0.035 and 0.437 ± 0.047, respectively. Both formulations spontaneously formed nanometric droplets upon aqueous dispersion. This confirms the self-nanoemulsifying behavior of the developed L-SNEDDS and PM-L-SNEDDS formulations.

#### 3.4.2. pH Measurement

The measured pH values for the plain and pH-modulated liquid SNEDDS were 5.31 ± 0.09 and 6.83 ± 0.10, respectively. The observed increase in pH is attributed to the addition of sodium carbonate. The increase in the microenvironmental pH is expected to enhance the drug-loading capacity of atorvastatin calcium and glibenclamide. This is because the pKa values of atorvastatin calcium and glibenclamide are 4.5 and 5.3, respectively [33,34] which are both lower than the pH of the pH-modulated liquid SNEDDS (6.83). Thus, the solubility of both drugs in the SNEDDS formulation is expected to be enhanced owing to their presence in ionized form.

#### 3.4.3. FTIR

**Figure 5:**
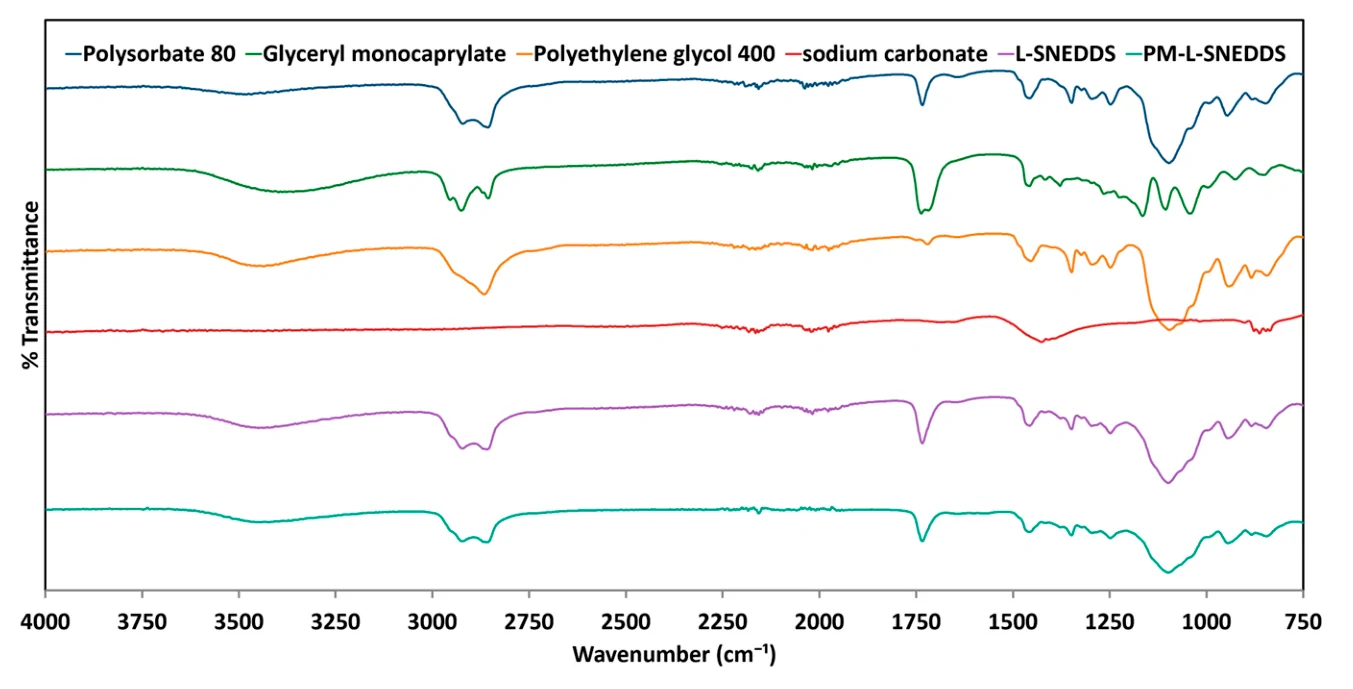
FTIR spectra of polysorbate 80, glyceryl monocaprylate, polyethylene glycol 400, sodium carbonate, L- SNEDDS, and PM-L-SNEDDS.

Figure 5 shows the FTIR spectra of both the liquid and pH-modulated SNEDDS formulations, along with their individual ingredients (Polysorbate 80, Glyceryl monocaprylate, polyethylene glycol 400, and sodium carbonate). The characteristic broad O–H band around 3450 cm^−1^ in SNEDDS formulations resulted from the reported hydroxyl groups of glyceryl monocaprylate, polyethylene glycol 400, and polysorbate 80. Moreover, the observed ester C=O band around 1735 cm^−1^ originated from the ester linkage in glyceryl monocaprylate and polysorbate 80. In addition, the absorption band at approximately 1100 cm^−1^ was assigned to the C–O–C ether stretching vibration. This is primarily arising from the polyoxyethylene chains of polyethylene glycol 400 and Polysorbate 80. The preservation of ingredients peaks without the emergence of new absorption bands indicates the absence of chemical interactions between the SNEDDS components. The disappearance of the characteristic carbonate absorption peak of sodium carbonate in the pH-modulated formulation spectrum can be attributed to its low loading concentration (5 mg/g). The present results confirm that the incorporation of sodium carbonate as a pH-modulating agent did not induce any chemical interaction or structural modification of the SNEDDS formulation.

### 3.5. Drug Loading Capacity

**Figure 6:**
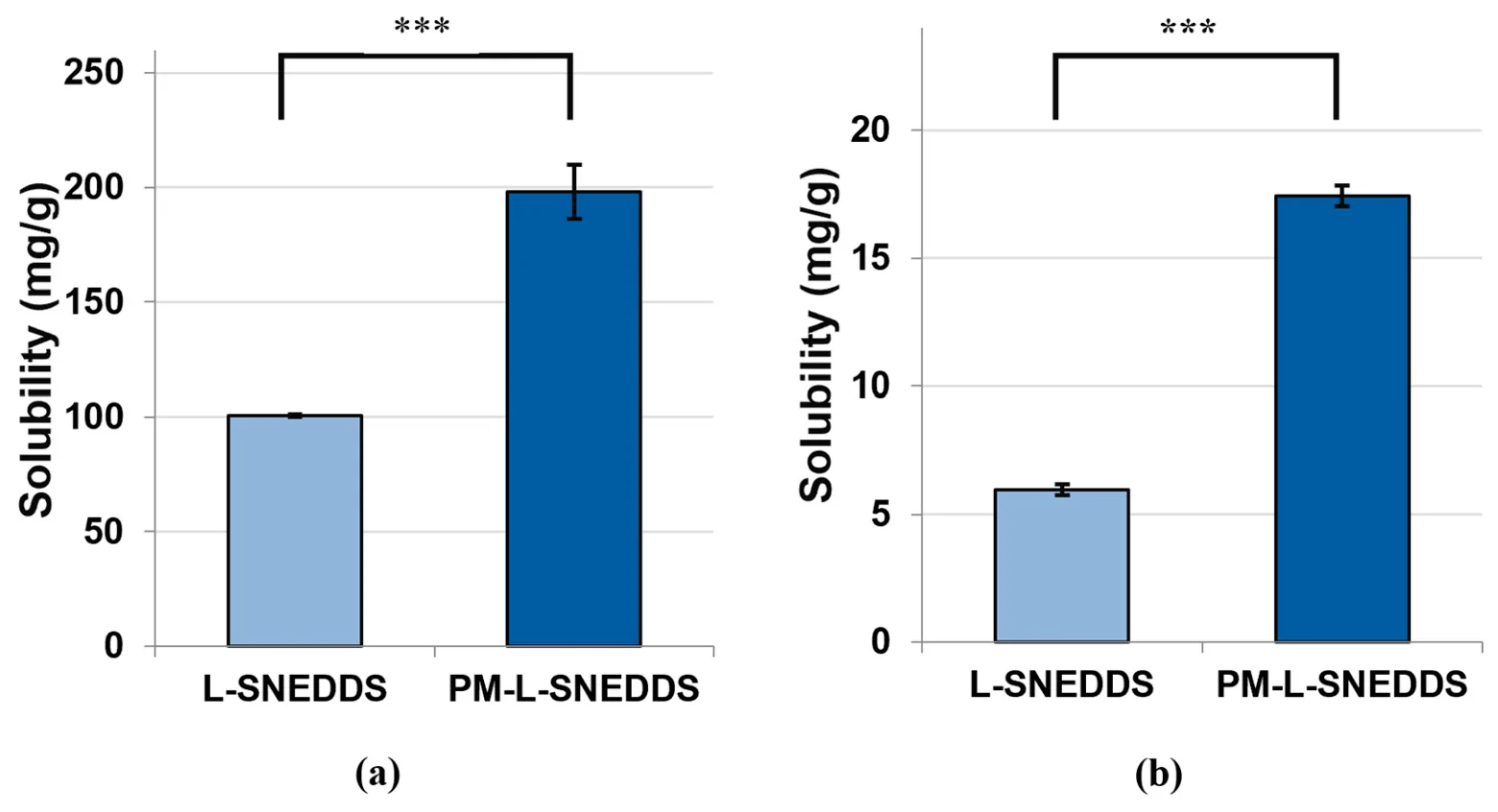
Drug-loading capacity of (**a**) atorvastatin calcium and (**b**) glibenclamide in plain liquid SNEDDS (L-SNEDDS) versus pH-modulated liquid SNEDDS (PM-L-SNEDDS). Data are presented as the mean ± SD (*n* = 3). Statistical significance was assessed using an unpaired Student’s *t*-test (*** *p* < 0.001).

The pH-modulating agent’s impact on the drug-loading capacity of both drugs was assessed in both formulations, and the data are presented in Figure 6. The present results showed that the solubility of glibenclamide and atorvastatin calcium in the liquid SNEDDS formulation was 5.97 ± 0.22 and 100.78 ± 0.58 mg/g, respectively. The integration of a pH-modulating agent into a liquid SNEDDS formulation increased the solubility of both drugs by approximately 3.0- and 2.0-fold, respectively. The observed increase in the solubility of both drugs is attributed to their weak acidity: the addition of sodium carbonate raises the pH of the liquid SNEDDS formulation above the drugs’ pKa and enhances the ionization of atorvastatin calcium and glibenclamide. Moreover, the enhanced drug loading could be further increased by in situ salt formation between the ionized drug and the alkalizing counterion [35]. The formed ionized drug could be associated with the hydrophilic surfactant (Polysorbate 80) and cosolvent (polyethylene glycol 400) components of the SNEDDS formulation.

### 3.6. Impact of pH-Modulating Agent on Dissolution Profile

**Figure 7:**
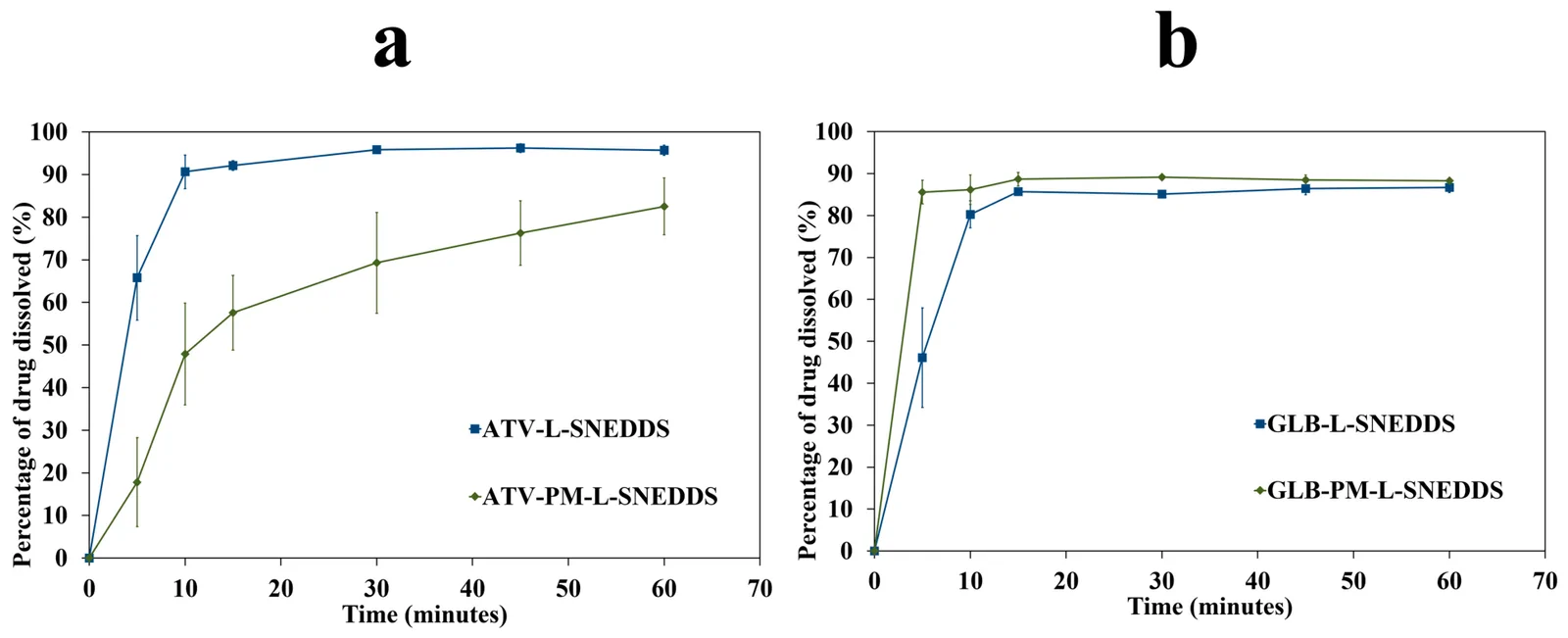
In vitro dissolution profiles of (**a**) atorvastatin calcium and (**b**) glibenclamide from liquid SNEDDS (L-SNEDDS) and pH-modulated liquid SNEDDS (PM-L-SNEDDS) formulations.

Figure 7 shows the dissolution profile of atorvastatin calcium and glibenclamide loaded within liquid and pH-modulated liquid SNEDDS formulations. Both formulations demonstrated favorable dissolution performance, with more than 80% of atorvastatin dissolved at the end of the experiment (Figure 7a). However, the PM-L-SNEDDS formulation displayed a notably slower initial dissolution rate compared to the L-SNEDDS formulation. This observed reduction in dissolution kinetics of atorvastatin calcium from the PM-L-SNEDDS formulation can be attributed to several factors. Firstly, the PM-L-SNEDDS formulation has a two-fold higher concentration than the conventional liquid SNEDDS formulation. This increased the formulation’s viscosity, hindering its rapid dispersion in the dissolution medium. Secondly, a high drug concentration adversely affects the formulation-to-drug ratio, thereby diminishing the relative proportion of dispersed nanoemulsion droplets available per unit of drug. Despite this initial lag, both formulations achieved satisfactory dissolution profiles, indicating their potential suitability for the oral delivery of poorly water-soluble drugs.

Regarding formulations loaded with glibenclamide, more than 80% of the drug was dissolved within 10 min, and this level was maintained throughout the experiment (Figure 7b). In contrast to atorvastatin calcium, the PM-L-SNEDDS formulation showed a relatively rapid initial dissolution rate of glibenclamide compared to the L-SNEDDS formulation, even with approximately 3.0-fold higher drug loading. The observed enhancement in dissolution behavior of the pH-modulated formulation can be attributed to the following reasons. Firstly, the incorporation of sodium carbonate as a pH-modulating agent created a localized alkaline microenvironment upon contact with the dissolution medium. This is expected to increase the solubility of weakly acidic glibenclamide through its ionization and dissolution rate. Secondly, the lower therapeutic dose of glibenclamide compared to atorvastatin calcium resulted in a greater number of dispersed nanoemulsion droplets available for drug solubilization.

### 3.7. Physicochemical Characterization of Solid SNEDDS Formulations

#### 3.7.1. SEM

**Figure 8:**
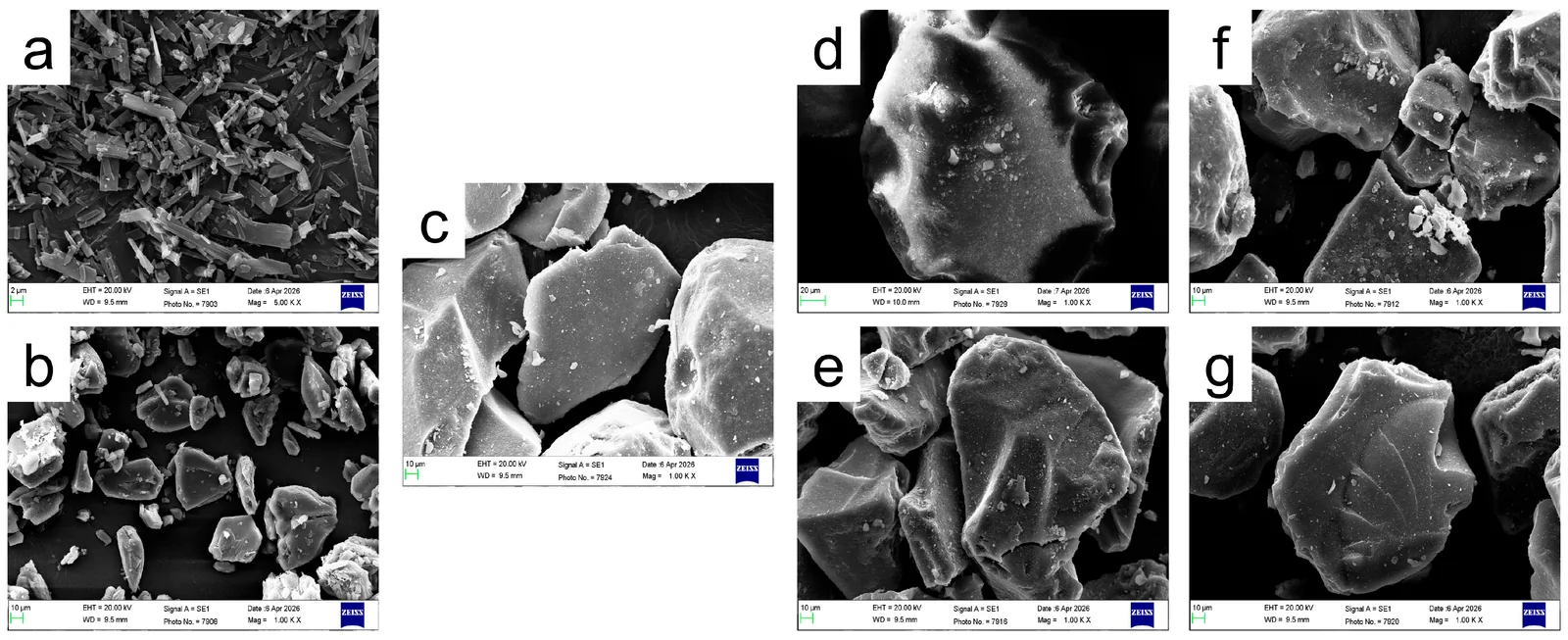
SEM images of (**a**) atorvastatin calcium, (**b**) glibenclamide, (**c**) Syloid adsorbent, (**d**) ATV-S-SNEDDS, (**e**) GLB-S-SNEDDS, (**f**) ATV-PM-S-SNEDDS, and (**g**) GLB-PM-S-SNEDDS.

Figure 8 presents SEM images of both drugs (atorvastatin calcium and glibenclamide), Syloid adsorbent, and the solidified SNEDDS formulations (ATV-S-SNEDDS, ATV-PM-S-SNEDDS, GLB-S-SNEDDS, and GLB-PM-S-SNEDDS). Raw atorvastatin calcium (Figure 8a) exhibited characteristic rod-shaped crystalline particles, which are in alignment with the prismatic crystal habit of its trihydrate polymorph [23]. In contrast, the raw glibenclamide SEM images (Figure 8b) showed irregular crystalline particles, which agreed with a previously reported study [36]. The Syloid adsorbent particles (Figure 8c) exhibited a lamellar shape with a distinctive porous structure [37]. The ATV-S-SNEDDS (Figure 8d), GLB-S-SNEDDS (Figure 8e), ATV-PM-S-SNEDDS (Figure 8f), and GLB-PM-S-SNEDDS (Figure 8g) formulations showed successful adsorption of the SNEDDS formulation onto the adsorbent surface. Furthermore, no distinct crystalline drug particles were visible on the adsorbent surface in any of the solidified formulations. Moreover, solid SNEDDS images confirmed the successful adsorption of SNEDDS within the porous structure of the Syloid adsorbent, as indicated by the absence of oil globules [38]. Furthermore, the vanishing of the distinguishing crystalline appearance of both drugs suggests that they could be present in solubilized form within the porous Syloid adsorbent [39]. Consequently, FTIR and PXRD were performed to examine the solid-state properties of both drugs in the SNEDDS.

#### 3.7.2. FTIR

**Figure 9:**
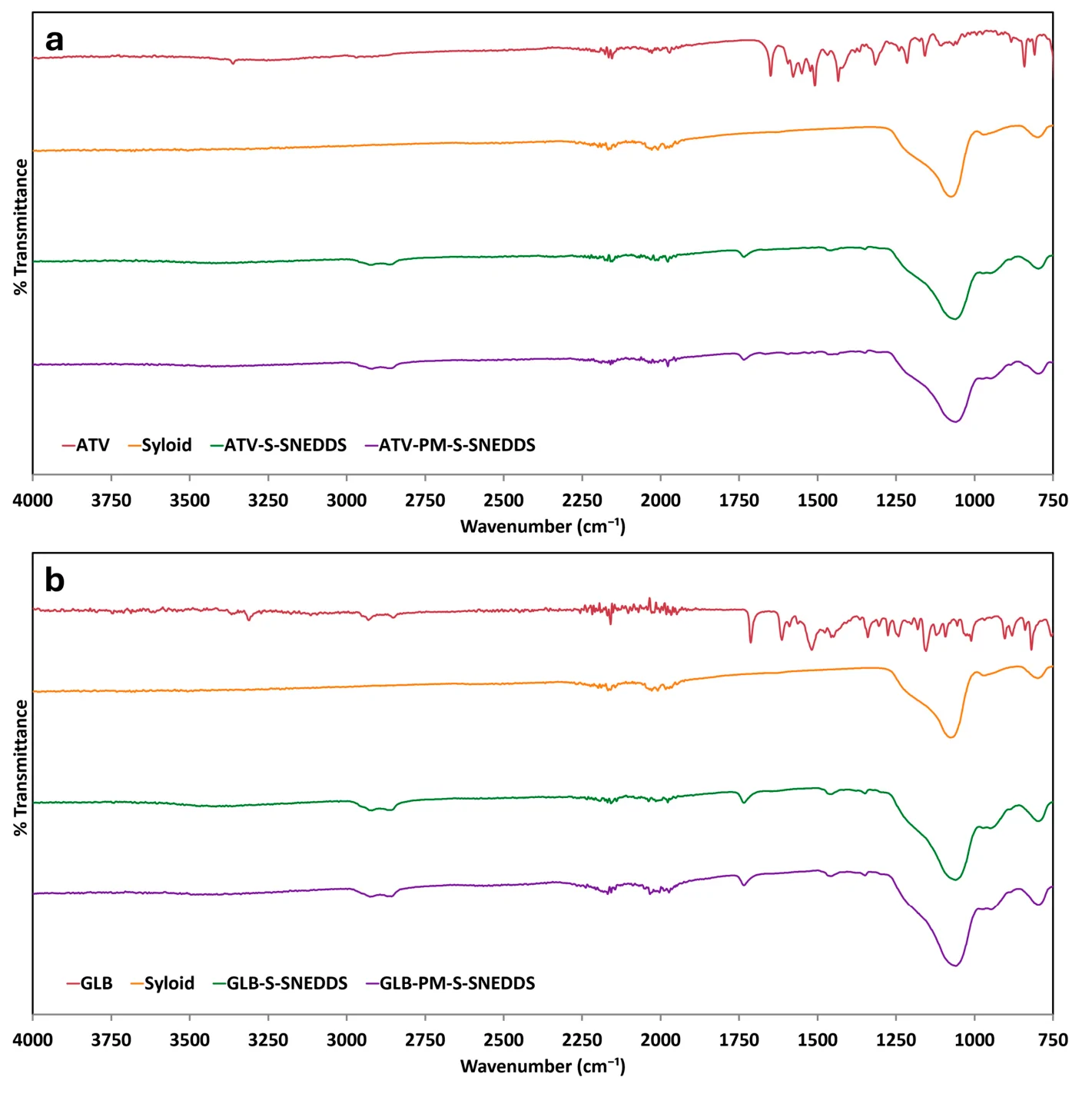
FTIR spectra of solidified SNEDDS formulations. (**a**) Atorvastatin calcium, Syloid, ATV-S-SNEDDS, and ATV-PM-S-SNEDDS. (**b**) Glibenclamide, Syloid, GLB-S-SNEDDS, and GLB-PM-S-SNEDDS.

Figure 9 presents the FTIR spectra of the solidified formulations, compared with the raw drugs and the Syloid adsorbent. The raw atorvastatin calcium spectrum displayed characteristic absorption peaks at 3363, 1651, 1578, and 1216 cm^−1^, corresponding to N–H stretching vibration, amide carbonyl (C=O) stretching vibration, N–H bending, and C–F stretching. On the other hand, the raw glibenclamide spectrum exhibited carbonyl (C=O) stretching vibration at 1714 cm^−1^ and the N–H bending vibration at 1520 cm^−1^. The FTIR spectrum of Syloid adsorbent displayed the characteristic three-band fingerprint of amorphous silica. The dominant broad absorption band at approximately 1079 cm^−1^ was assigned to the asymmetric Si–O–Si stretching vibration. The Si–OH silanol stretching vibration was observed at 972 cm^−1^, while the symmetric Si–O–Si stretching appeared at 800 cm^−1^.

The FTIR spectra of the solidified SNEDDS formulations (ATV-S-SNEDDS, ATV-PM-S-SNEDDS, GLB-S-SNEDDS, and GLB-PM-S-SNEDDS) displayed a spectral profile dominated by the Syloid. Moreover, SNEDDS excipient bands were observed in the prepared formulations. On the other hand, the characteristic bands of atorvastatin calcium and glibenclamide were absent in the solidified formulation spectra. This could be ascribed to the solubilization of both drugs in the SNEDDS formulation and their molecular dispersion within the SNEDDS formulation. To investigate the molecular state of atorvastatin calcium and glibenclamide within the formulation, PXRD was performed on the solidified formulations.

#### 3.7.3. PXRD

**Figure 10:**
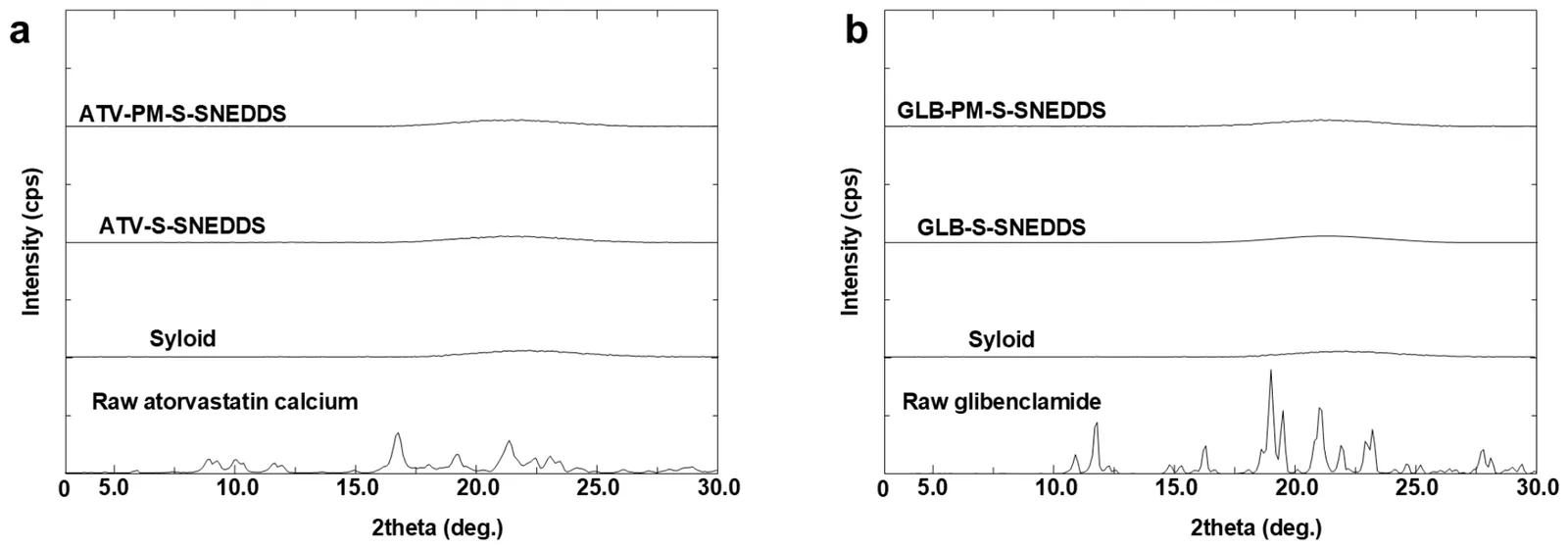
PXRD diffractograms of (**a**) raw atorvastatin calcium, Syloid, ATV-S-SNEDDS, and ATV-PM-S-SNEDDS; and (**b**) raw glibenclamide, Syloid, GLB-S-SNEDDS, and GLB-PM-S-SNEDDS.

Figure 10 shows the diffraction profile of both drugs (atorvastatin calcium and glibenclamide), Syloid adsorbent, and the solidified SNEDDS formulations (ATV-S-SNEDDS, ATV-PM-S-SNEDDS, GLB-S-SNEDDS, and GLB-PM-S-SNEDDS). The diffractogram of raw atorvastatin calcium exhibited numerous sharp crystalline diffraction peaks, with characteristic two sharp peaks at 16.8° and 21.4°. In addition, raw glibenclamide displayed a highly crystalline diffraction pattern with numerous sharp crystalline peaks at 11.8°, 19.0°, 21.0°, and 23.2°. However, the Syloid diffraction pattern showed a complete absence of characteristic peaks due to the adsorbent’s amorphous and porous nature. The diffraction patterns of all solidified SNEDDS formulations (ATV-S-SNEDDS, ATV-PM-S-SNEDDS, GLB-S-SNEDDS, and GLB-PM-S-SNEDDS) showed no detectable crystalline peaks for either atorvastatin calcium or glibenclamide. The molecular-level solubilization of atorvastatin calcium and glibenclamide in the liquid SNEDDS prior to adsorption prevents their conversion to the crystalline state. This agrees with the observed absence of crystalline peaks and absence of crystalline drug particles in the SEM images.

### 3.8. Impact of Solidification on Dissolution Profile

**Figure 11:**
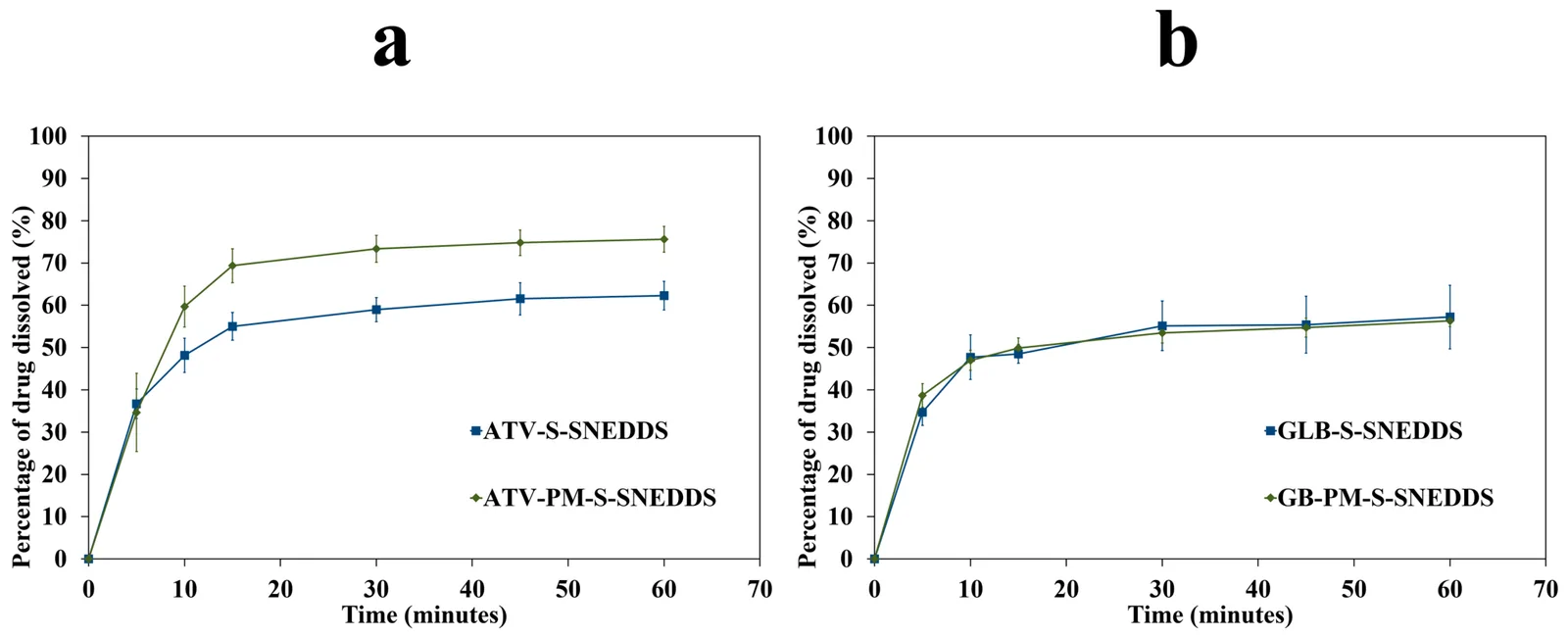
In vitro dissolution profiles of (**a**) atorvastatin calcium and (**b**) glibenclamide from solid SNEDDS (S-SNEDDS) and pH-modulated solid SNEDDS (PM-S-SNEDDS) formulations. Data are presented as mean ± standard deviation (*n* = 3).

The prepared formulations were solidified using Syloid adsorbent to investigate the combination effect of pH modulation and solidification on formulation performance. The in-vitro dissolution profile of atorvastatin calcium and glibenclamide from solid and pH-modulated solid SNEDDS formulations is presented in Figure 11. The current results showed that the pH-modulated agent enhanced the dissolution of atorvastatin calcium compared to the plain formulation. On the other hand, the pH-modulated agent did not significantly affect the dissolution profile of glibenclamide.

Solidification of the liquid SNEDDS onto Syloid can slow drug release relative to the liquid formulation. This is owing to physical interactions between the loaded drug and the adsorbent. Mesoporous silica carriers possess a defined number of surface silanol groups (Si–OH) that function as hydrogen-bond donors [40]. These functional groups can interact with drug molecules loaded into the SNEDDS formulation [41]. Therefore, during the solidification of liquid SNEDDS onto the Syloid adsorbent, the loaded drug could remain dissolved in the SNEDDS liquid within the pore cavity or interact directly with the silanol groups.

**Figure 12:**
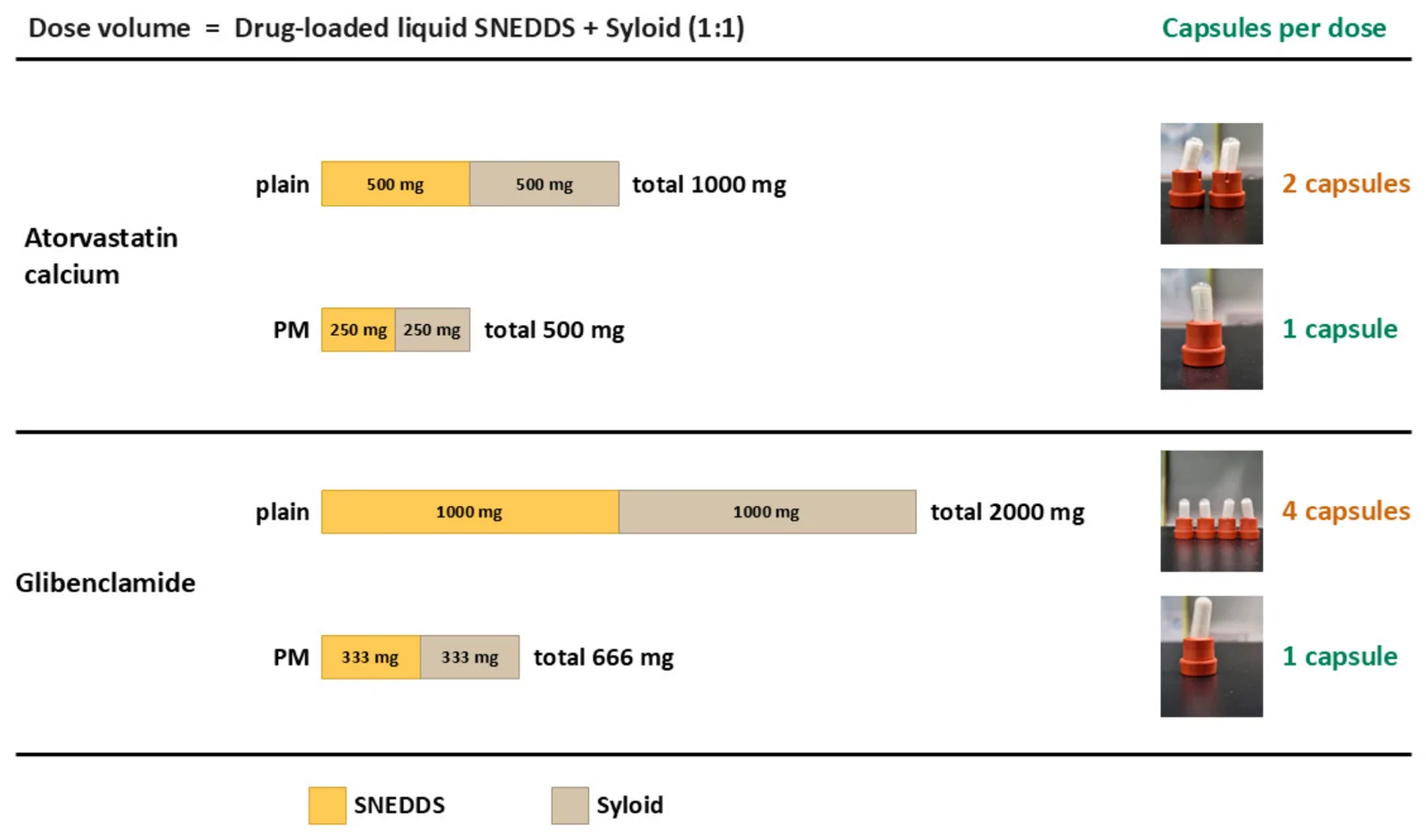
Reduction in dose volume and capsule number by pH modulation. For each drug, bars show the total dose volume as liquid SNEDDS (gold) plus Syloid carrier (grey) at a 1:1 ratio, with the corresponding capsules shown alongside. Higher drug loading lowers the liquid SNEDDS and co-adsorbed Syloid per dose. This reduced atorvastatin calcium from two capsules to one and glibenclamide from four to one.

A practical limitation of adsorption-based solidification is the increase in dose volume. This is because the liquid SNEDDS must be loaded onto a solid carrier (Syloid), which is added here at a fixed 1:1 ratio. Because Syloid has a low bulk density, it limits the total powder volume that can be filled into capsules. By raising the drug-loading capacity of the SNEDDS, pH modulation reduced the amount of SNEDDS and the amount of co-adsorbed Syloid required to deliver each dose. This decreases both the total dose volume and the number of capsules (Figure 12). For atorvastatin calcium (40 mg), loading increased ~2-fold (from 8 to 16% *w*/*w*), halving the SNEDDS required from 500 to 250 mg and the total powder from 1000 to 500 mg. This reduced the dose from two capsules to one. For glibenclamide (5 mg), loading increased approximately 3.0-fold (from 0.5 to 1.5% *w*/*w*), lowering the SNEDDS from 1000 to 333 mg and the total powder from 2000 to 666 mg, reducing the dose from four capsules to a single capsule.

**Figure 13:**
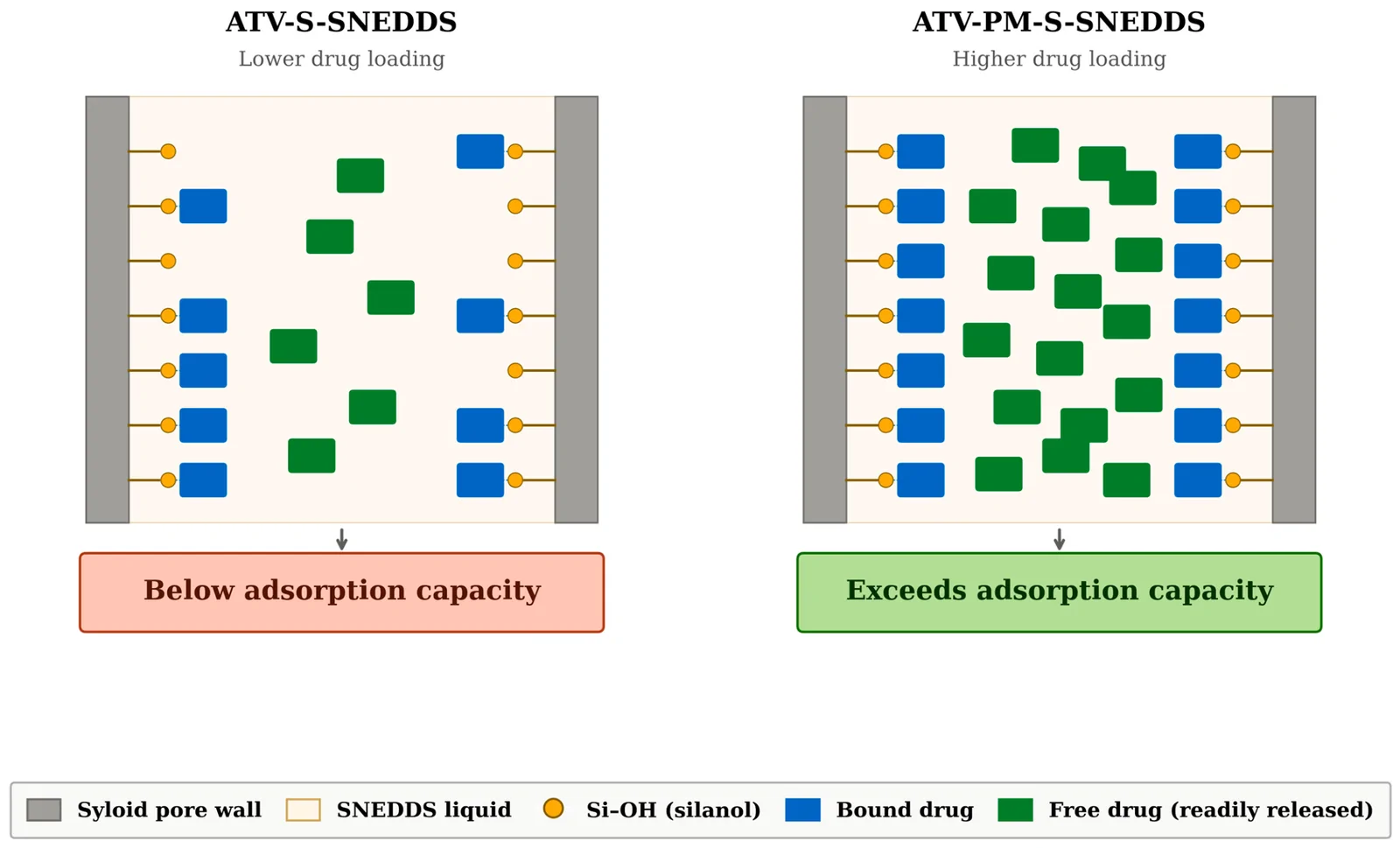
Schematic illustration of the proposed silanol saturation mechanism for atorvastatin calcium in PM-S-SNEDDS (**right**) formulation against S-SNEDDS (**left**).

Along with the observed reduction in dosage volume with atorvastatin calcium (Figure 12), PM-S-SNEDDS offers additional advantages by enhancing the dissolution profile of atorvastatin calcium compared to the S-SNEDDS formulation (Figure 11). This could be explained through the dose-dependent silanol saturation effect. The achieved 2-fold increase in atorvastatin calcium drug loading exceeds the adsorbent’s adsorption capacity (Figure 13). Consequently, the excess drug molecules that are not bound to silanol groups remain in free form within the pore interior and are readily accessible to dissolution media upon contact with the aqueous environment.

## 4. Conclusions

This work demonstrates that pH modulation with sodium carbonate is an effective strategy to overcome the two limitations of adsorption-based solid SNEDDS. These include low drug loading and high dosage volume. Raising the pH of the formulation microenvironment above the pKa of the weakly acidic model drugs increased the loading of atorvastatin calcium and glibenclamide by approximately 2.0- and 3.0-fold, respectively. This enabled the reduction of the capsule requirements from multiple units to a single capsule for both drugs. The used characterization techniques confirmed that the carbonate addition did not alter the formulation chemistry and that the drugs were adsorbed within Syloid without detectable crystallinity. pH-modulated SNEDDS represents a simple and cost-effective approach that simultaneously addresses both the limited drug solubility and dose-volume limitations. This is expected to improve the pharmaceutical applicability of the adsorption-based solidification approach.

## Figures and Tables

**Table 1:** Summary of UPLC Chromatographic Conditions.

Parameter	Glibenclamide	Atorvastatin Calcium
Mobile Phase	Acetonitrile: 0.1% Formic acid (46.9:53.1, *v*/*v*)	0.1% Formic acid: 10 mM Ammonium formate: Acetonitrile (10:45:45, *v*/*v*/*v*)
Flow Rate (mL/min)	0.3	0.4
Column Temperature (°C)	38.8	30.0
Detection Wavelength (nm)	228	245
Calibration Range (µg/mL)	0.5–20.0	5.0–50.0

**Table 2:** Measured transmittance values for polysorbate surfactants and glyceryl monocaprylate mixtures.

Surfactant	Transmittance (%)
Polysorbate 85	50.87 ± 0.31
Polysorbate 80	82.83 ± 0.45
Polysorbate 60	79.60 ± 0.10
Sorbitan monolaurate	NA
sorbitan monooleate	NA

## Data Availability

Data is available in the manuscript.
